# Blood cultures in ambulatory outpatients

**DOI:** 10.1186/1471-2334-5-35

**Published:** 2005-05-17

**Authors:** Kevin B Laupland, Deirdre L Church, Daniel B Gregson

**Affiliations:** 1 Departments of Medicine, Centre for Anti-microbial Resistance and Infectious Diseases Research Group, University of Calgary, Calgary Health Region, and Calgary Laboratory Services, Calgary, Alberta, Canada; 2 Critical Care Medicine, Centre for Anti-microbial Resistance and Infectious Diseases Research Group, University of Calgary, Calgary Health Region, and Calgary Laboratory Services, Calgary, Alberta, Canada; 3 Pathology and Laboratory Medicine, Centre for Anti-microbial Resistance and Infectious Diseases Research Group, University of Calgary, Calgary Health Region, and Calgary Laboratory Services, Calgary, Alberta, Canada

## Abstract

**Background:**

Blood cultures are a gold standard specific test for diagnosing many infections. However, the low yield may limit their usefulness, particularly in low-risk populations. This study was conducted to assess the utility of blood cultures drawn from ambulatory outpatients.

**Methods:**

Blood cultures drawn at community-based collection sites in the Calgary Health Region (population 1 million) in 2001 and 2002 were included in this study. These patients were analyzed by linkages to acute care health care databases for utilization of acute care facilities within 2 weeks of blood culture draw.

**Results:**

3102 sets of cultures were drawn from 1732 ambulatory outpatients (annual rate = 89.4 per 100,000 population). Significant isolates were identified from 73 (2.4%) sets of cultures from 51 patients, including *Escherichia coli *in 18 (35%) and seven (14%) each of *Staphylococcus aureus *and *Streptococcus pneumoniae*. Compared to patients with negative cultures, those with positive cultures were older (mean 49.6 vs. 40.1 years, p < 0.01), and more likely to subsequently receive care at a regional emergency department, outpatient antibiotic clinic, or hospital (35/51 vs. 296/1681, p < 0.0001). Of the 331 (19%) patients who received acute care treatment, those with positive cultures presented sooner after community culture draw (median 2 vs. 3 days, p < 0.01) and had longer median treatment duration (6 vs. 2 days, p < 0.01).

**Conclusion:**

Blood cultures drawn in outpatient settings are uncommonly positive, but may define patients for increased intensity of therapy. Strategies to reduce utilization without excluding patients with positive cultures need to be developed for this patient population.

## Background

Positive blood cultures are considered a gold standard specific test for diagnosing and managing patients with bacterial infections. However, with the exception of a few infectious foci such as endocarditis or meningitis, their low sensitivity usually limits their diagnostic utility. Several hospital-based studies have indicated that blood cultures are typically positive in less than 10% of bacterial pneumonias, soft tissue infections, and urinary tract infections and as a result their performance may not be cost effective [1-13]. One study in the 1970s identified that the yield of blood cultures obtained in an emergency department was lower in those selected for ambulatory as compared to hospital care [14]. However, such a distinction may not be relevant today as a result of healthcare restructuring over the past decade that has seen a shift toward care of sicker patients in the community. There are no studies published that have investigated the utility of blood cultures obtained from community-based outpatient settings.

This study was undertaken to comprehensively evaluate the occurrence of sampling, and rate and predictors of positive blood cultures obtained from all community-based outpatient collection sites in the Calgary Health Region. We also sought to determine if positive blood culture results were associated with subsequent use of acute care facilities for treatment, by performing a database linkage to all emergency departments, hospitals, and outpatient parenteral antibiotic clinics in this large Canadian region.

## Methods

### Patient population

The Calgary Health Region (CHR) is a fully integrated, publicly funded health system that provides virtually all medical and surgical care to the one million residents of the cities of Calgary and Airdrie and approximately 20 nearby small towns, villages, and hamlets. In the CHR, Calgary Laboratory Services (CLS) receives all specimens submitted for blood culture testing from all acute care hospitals and 24 community collection sites in the region [15]. This study included all blood samples submitted for culture from outpatient community collection sites in the CHR from January 1, 2001 to December 31, 2002. Samples submitted from all emergency departments, hospitals, and hospital-based clinics were excluded. This study was reviewed by the Conjoint Health Research Ethics Board at the University of Calgary and Calgary Health Region.

### Study protocol

A laboratory-based cohort design with linkage to hospital administrative databases was utilized. All blood cultures submitted from community-based collection sites in the CHR during the study period were identified by use of the Cerner PathNet Classic version 306 (Kansas City, MO) database at CLS. Basic demographic information including age, gender, and community collection site location and the organism cultured were exported. In order to determine whether patients subsequently accessed an acute care service in the CHR within two weeks of blood culture draw, linkages to two regional databases were performed. The Health Information Services database that registers all emergency department and acute care hospital admissions and the Home Parenteral Therapy Program (HPTP) database that registers all patients treated with outpatient intravenous antibiotic therapy in the CHR were queried for all patient encounters [16].

For all patients receiving acute care services, the type, location, and duration of care and the most responsible diagnosis was recorded. Because patients may be treated through more than one acute care service, such as assessment in an emergency department and subsequent admission to hospital, acute care encounters were classified in a hierarchal fashion. These included emergency visit only, HPTP clinic assessment (with or without emergency visit), and admission to hospital (with or without emergency and/or HPTP clinic treatment). Data from each of the three databases was exported to Excel 2000 (Microsoft Corp., Redmond, WA) and merged using Access 2000 (Microsoft Corp.).

### Laboratory procedures and definitions

All blood was cultured at CLS using the BacT/Alert automated instrument (Organon Teknika, Durham, NC). A blood culture set consisted of an aerobic/anaerobic bottle pair of BacT/Alert FAN bottles obtained from a single draw [17]. Inoculated bottles were immediately placed in the instrument, incubated at 37°C and continuously read for growth in BacT/Alert 2-D cabinets. A significant isolate was defined as the growth of a pathogenic organism from at least one set of blood cultures. At least two positive sets of blood cultures within a 48 hour period were required to classify common contaminants including coagulase negative staphylococci, viridans group streptococci, or *Bacillus*, *Corynebacterium*, or *Propionibacterium species *as significant. Significant isolates were identified and tested for antimicrobial susceptibility according to National Committee for Clinical Laboratory Standards guidelines. Antibiotic resistant organisms were defined as methicillin resistant *Staphylococcus aureus*, vancomycin resistant *Enterococcus faecalis *or *faecium*, *Streptococcus pneumoniae *with reduced susceptibility to penicillin, or any Gram-negative organisms resistant to ciprofloxacin, tobramycin, gentamicin, ceftazidime, or carbapenems.

### Analysis

The base dataset that included all culture draws was used to describe blood culture sampling rates in the region. If a patient had multiple blood cultures submitted they were deemed to represent distinct "episodes" if different sets were drawn greater than two calendar days apart. In the assessment of demographic and outcome information, the analysis was restricted to include only a given patient's first episode. This was performed to avoid analysis of correlated measures due to repeat patient presentations/episodes.

All analyses were performed using Stata version 7.0 (Stata Corp., College Station, TX). Variables were assessed using histograms prior to analysis to identify outlying data points and to assess underlying distribution. Means with standard deviations (SD) were used to describe normally or near normally distributed variables and medians with interquartile ranges (IQR) for non-normally distributed variables. Differences in proportions were compared using Fisher's exact test. Means were compared with the Student *t *test and medians using the Wilcoxon Rank-sum test. Incidence and relative risk (RR) calculations with exact 95% confidence intervals (CI) were performed as previously described [18]. For these calculations we assumed that patients were CHR residents if patients had Alberta health care numbers and cultures were drawn at CHR collection sites.

## Results

### Occurrence of sampling

**Figure 1:**
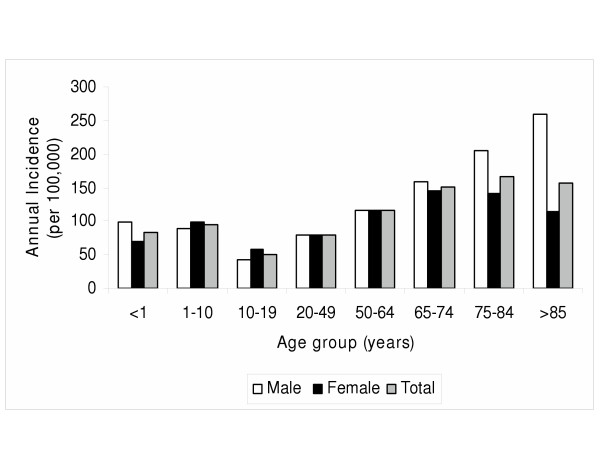
Annual age and gender related blood culture sampling incidence at community-based outpatient collection sites, Calgary Health Region, Alberta, Canada (2001 and 2002).

During the study period, 3102 sets of blood cultures were drawn from 1732 patients for an annual rate of sampling of 89.4 per 100,000-health region residents. A single blood culture only was taken on 371 patients (21%). The rest had two or more sets of blood cultures drawn including 79 (5%) with three sets, 62 with four sets, 18 with five sets, and 14 with six sets. Two patients had seven sets and one patient each had eight to 14 sets submitted. During 2001 and 2002 a total of 1567 and 1535 sets of cultures were submitted from 895 and 837 patients for annual incidences of culturing of 93.4 and 85.6 per 100,000 population (p = 0.04), respectively. The mean ± SD age of patients was 40.2 ± 22.6 years overall and there was substantial variability in performance of first episodes of blood culturing based on age as shown in figure 1. There was a similar rate of culturing among males and females with annual incident culturing rates of 89.9 and 91.8 per 100,000 (p = 0.3), respectively. However, among patients 65 years or older, males were more likely to be sampled than females (179.9 vs. 139.6 per 100,000; RR = 1.3; 95% CI, 1.01 to 1.64; p = 0.02).

### Rate and etiology of positive cultures

Of the total 3102 sets of blood cultures, 108 (3.5%) grew an organism and only 74 (2.4%) of these were deemed to represent significant isolates. The 34 (1.1%) contaminants included coagulase negative staphylococci in 25 sets, two sets each of *Micrococcus species*, viridians group streptococci, and *Bacillus species*, and one set each of gram positive bacilli, *Streptococcus mitis*, and coryneform bacilli. Significant isolates were identified from 73 sets of cultures from 51 patients at first episode and these were most commonly *Escherichia coli *in 18 (35%) and seven (14%) each of *Staphylococcus aureus *(co-isolated with group A streptococcus in one case and streptococcal species in another) and *Streptococcus pneumoniae *patients as shown in table 1. Only one patient had an infection on a second or subsequent episode of blood culturing. This 76 year old man had an *Escherichia coli *bacteremia at a second culturing episode two months after he had a negative set of cultures drawn.

**Table 1:** Infectious etiology among 51 patients identified with community-based blood cultures first episodes, Calgary Health Region, Alberta, Canada (2001 and 2002).

**Species**	**Occurrence **(n = 54*)
*Escherichia coli*	18 (35%)
*Staphylococcus aureus*	7 (14%)
*Streptococcus pneumoniae*	7 (14%)
Group A streptococcus	4 (8%)
*Klebsiella pneumoniae*	3 (6%)
Coagulase negative staphylococci	3 (6%)
*Dialister pneumosintes*	1 (2%)
*Bacteroides fragilis*	1
*Abiotrophia species*	1
*Neisseria meningitidis*	1
*Peptostreptococcus *species	1
*Proteus mirabilis*	1
*Pseudomonas aeruginosa*	1
*Salmonella species *group C2	1
*Streptococcus mitis *group	1
*Streptococcus milleri *group	1
*Streptococcus sanguis *group	1
*Streptococcus *species	1

Note: * Numbers do not add to 51 because three patients had polymicrobial infection; one each of *Staphylococcus aureus*/streptococcal species, *Staphylococcus aureus*/group A streptococcus, and *Escherichia coli*/*Bacteroides fragilis*.

Of the 24 patients with an aerobic Gram-negative rod bacteremia, a urine culture was not done in seven cases, had discordant (negative) results in seven cases, and was concordant (positive with identical isolate) in 10 cases. The one patient with an extended spectrum beta-lactamase producing *E. coli *had a concordant urine sample. The one patient with *Pseudomonas aeruginosa *bacteremia had no other cultures collected. All isolates of *S. aureus *were methicillin susceptible. In eight (16%) patients with positive cultures, the anaerobic bottle was independently positive and the organisms were all facultative anaerobes (*E. coli *in five and one each of *Klebsiella pneumoniae*, *Proteus mirabilis*, and *S. aureus*).

### Predictors and outcomes associated with positive blood cultures

A number of demographic, clinical, and outcome characteristics were associated with blood culture positivity and are shown in table 2. Patients with positive cultures were of significantly higher mean age (table 2) with those aged 50 years or more likely to have a positive culture as compared to younger patients (RR 1.6, 95% CI, 1.3 to 2.1, p < 0.01). Although only 252 (15%) patients had blood cultures submitted at the main high volume lab location, nearly one third (16/51) of all positives cultures were from that site (p < 0.01).

**Table 2:** Demographic characteristics and acute care management of patients with blood cultures submitted to outpatient collection sites, Calgary Health Region, Calgary, Alberta (2001 and 2002).

**Characteristic**	**Positive cultures **(n = 51)	**Negative cultures **(n = 1681)	**Total **(n = 1732)	P-value*
Mean age ± standard deviation (years)	49.6 ± 21.1	40.1 ± 22.6	40.3 ± 22.6	< 0.01
Male gender	30 (59%)	829 (49%)	859 (50%)	0.2
Collection at high volume (main) lab vs. other sites	16 (31%)	236 (14%)	252 (15%)	< 0.01
Acute care usage within two weeks of culture	35 (69%)	296 (18%)	331 (19%)	< 0.0001
Emergency department	9 (18%)	147 (9%)	156 (9%)	0.04
HPTP clinic	2 (4%)	15 (1%)	17 (1%)	0.08
Admitted to hospital	24 (47%)	134 (8%)	158 (9%)	< 0.0001
Median days of acute care therapy	6 (IQR = 2, 14; n = 35)	2 (IQR = 1, 6; n = 296)	2 (IQR = 1, 6; n = 331)	< 0.001
Median time (days) to acute care assessment	2 (IQR = 1, 2; n = 35)	3 (IQR = 2, 5; n = 296)	2 (IQR = 1, 5; n = 331)	< 0.01

Note: * p-value for comparison between those with positive and negative cultures using Fisher's exact test, Student's T test, and Wilcoxon Rank-sum test for comparison of proportions, means, and medians, respectively.

Of the cohort of 1732 patients who had a first episode of blood cultures submitted, 331 (19%) subsequently received acute care treatment within two weeks of culture draw. One hundred and fifty-six patients were seen at an emergency department only, 17 were treated via an HPTP clinic (with or without emergency visit), and 158 patients were admitted to hospital (with or without emergency and/or HPTP clinic care). Patients with positive blood cultures utilized acute care services at a significantly higher rate, presented earlier, and had longer treatment durations (table 2). The increased use of acute care services was largely due to a nearly six-fold increased admission rate among those with positive as compared to negative cultures (RR 5.9, 95% CI 4.2 to 8.2, p < 0.0001). Patients with positive cultures presented earlier to acute care services following culture draw from an outpatient collection site than those with negative cultures and this was primarily due to a significantly increased rate of presentation on the second calendar day after blood culture draw. Although there was no difference in the proportion presenting the same calendar day (10/35 vs. 74/296, p = 0.7), significantly more patients with positive cultures were seen within two calendar days (< 48 hours) from the point of culture draw (27/35 vs. 147/296, p < 0.01).

Based on emergency, HPTP clinic, or admissions data, the most common recorded primary diagnoses were apparently non-infectious (22%), suspected non-focal or viral infection (18%), and respiratory tract infection (18%) as shown in table 3. There was an overall significantly different proportional distribution of diagnoses among patients with positive as compared to negative blood cultures (p = 0.001). Patients with positive blood cultures were more likely to be diagnosed with a genitourinary (RR = 2.9, 95% CI, 1.5 to 5.7, p < 0.01) or cardiovascular (RR = 6.7, 95% CI, 1.9 to 24, p < 0.01) focus as compared to patients with negative cultures (table 3).

**Table 3:** Primary diagnoses among 331 patients assessed at acute care sites following performance of outpatient blood cultures, Calgary Health Region, Alberta, Canada (2001 and 2002).

**Primary diagnosis**	**Positive cultures **(n = 35)	**Negative cultures **(n = 296)	**Total **(n = 331)	P-value*
No focus/virus	6 (17)	55 (18)	61 (18%)	1.0
Non-infective	4 (11)	70 (23)	69 (22%)	0.1
Soft tissue	1 (3)	35 (11)	31 (10%)	0.1
Bone/joint	1 (3)	11 (4)	12 (4%)	1.0
Respiratory	5 (14)	55 (20)	60 (19%)	0.6
Gastrointestinal	4 (11)	38 (13)	40 (13%)	1.0
Genitourinary	9 (26)	26 (9)	33 (11%)	< 0.01
Vascular	4 (11)	5 (2)	9 (3%)	< 0.01
Central nervous system	1 (3)	1	2 (1%)	0.2

Note: * p-value for comparison between those with positive and negative cultures using Fisher's exact test.

## Discussion

This is the first study to comprehensively evaluate the utility of blood cultures drawn from community-based outpatients. We showed that the performance of blood cultures on outpatients is a relatively common practice in our region. This may in part be related to the considerable healthcare restructuring that has occurred in recent years with an increased emphasis on care of patients in the community. Our positivity rate of 2.4% is similar to that of 1.8% found by Sturmann et al [11] in patients discharged from the emergency room. However, we do not have comparative data from the prior era to demonstrate that possibility. It is noteworthy that although there was an increasing age specific rate of performance of blood cultures in our population, this practice included a broad range of patients from infants to the elderly. One collection site had a higher number of positive cultures. We suspect this may be due to this site serving the urban poor population of the region.

In addition to its novelty as the first outpatient study of the utility of blood cultures, this study is also notable for its comprehensiveness of case identification and assessment of acute care utilization. Since all routine blood cultures are processed by CLS in our region and they are worked up and reported routinely using the CLS computer system, the number of patients having outpatient blood cultures that may have been missed by our study is negligible. It is also unlikely that we missed significant patient encounters in acute care settings because we performed a linkage to all institutions in the region. This included 496,141 emergency department, 6,462 HPTP clinic, and 189,897 hospital admissions over the two-year study period.

An important finding of this study is that while the rate of outpatient blood culture positivity was very low (2.4%), when positive, these results appeared to have had a significant association with patient management. Patients with positive cultures were more likely to receive acute care treatment, present earlier after culture draw, and be treated longer than patients with negative results. However, it is not clear from this retrospective observational study whether patient's management changed as a result of the culture or whether the differences observed were related to an increased severity of disease associated with a positive culture. Most studies have found that the positive blood cultures rarely result in a change in patient management [4,5,11,13,19,20]. However, in more recent studies on patients discharged from emergency rooms in Spain [21] and Israel [22], over a third of patients with positive blood cultures required either an initiation or change in antimicrobial therapy.

A central issue surrounding the performance of blood cultures in community-based settings is whether they are cost effective. Several studies that have looked at the low yield of blood cultures in a number of clinical settings and have suggested poor cost effectiveness for this test especially when the rate of positivity is low [3-5,11]. Arguments against the use of blood cultures in these settings based on poor cost effectiveness, however, have to be balanced against the risk of using excessively broad or inadequate empiric treatment. Based on the cost of USD$50 per aerobic and anaerobic set of blood cultures as determined by Perl *et al *[5], approximately $75,000 per year (or $1014 per significant positive set of blood cultures) is required to provide outpatient blood culture services in our region. As noted previously, it is not clear whether these tests specifically led to improvements in patient management.

There may be opportunities to better utilize outpatient blood cultures in our region. First, in patients with a clinical focus of infection such as pyelonephritis or cellulitis, clinical features alone or with other routine cultures (such as urine culture) may be enough to administer appropriate treatment. In our positive cohort, seven cases of urinary tract infections had positive blood cultures and negative urine cultures. This is a higher rate of discordance than in prior studies [8,12,19], which we feel is a consequence of antibiotic therapy before the collection of urine cultures. We agree with other investigators [8,12,23] that urine culture prior to antibiotics should be the primary diagnostic test with blood cultures being used selectively. Second, the use of an anaerobic in addition to aerobic bottle appears to provide relatively little added information and may not be required routinely. The anaerobic bottle was the only positive bottle in 8/1732 (0.5%) patients cultured. In 5/6 of these cases (where information was available) the diagnosis was urinary tract infection, and the etiology may have been available from urine cultures. In the remaining case, the isolate was *S. aureus *from a patient with a bone/joint source of infection. We therefore agree with Morris et al [24] that anaerobic blood cultures can be used selectively.

Finally, it is unclear whether blood culture results lead physicians in the region to reduce broad spectrum or inappropriate antibiotic usage. Although we are unable to address the practice in the region based on our study, several reports have suggested that physicians do not commonly alter their management accordingly [3,25]. Further prospective studies are required to better define means of identifying patients that may benefit most from the performance of outpatient blood cultures, including the use of clinical scoring systems[26,27] and/or screening tests [28-30].

## Conclusion

This study documents the use of blood cultures obtained from outpatients in community-based settings. Significant positive results are rare (only 2.4%). Patients with positive cultures do however, access acute care services more frequently than those with negative cultures and are more likely to be admitted to hospital. Further studies in community-based populations are required to define which patients are likely to benefit from blood cultures in this setting.

## Competing interests

The authors have not received any funding in the past five years from any organizations that may in any way gain or lose financially from the publication of this manuscript, either now or in the future.

None on the authors hold any stocks or shares in an organization that may in any way gain or lose financially from the publication of this manuscript.

There are no patents pending related to the content of this manuscript.

The authors do not have any non-financial competing interests to declare in relation to this manuscript.

## Authors' contributions

KL, DG, and DC were all involved in the study design. DG performed data extraction and KL developed database linking with regional data. KL performed data analysis and KL wrote the initial draft of this paper. DG and DC provided input into subsequent drafts and iterations of this manuscript. All authors read and approved the final manuscript.

## Pre-publication history

The pre-publication history for this paper can be accessed here:



## References

[B1] Shah SS, Alpern ER, Zwerling L, McGowan KL, Bell LM (2003). Risk of bacteremia in young children with pneumonia treated as outpatients. Arch Pediatr Adolesc Med.

[B2] Ikaheimo R, Siitonen A, Karkkainen U, Mustonen J, Heiskanen T, Makela PH (1994). Community-acquired pyelonephritis in adults: characteristics of E. coli isolates in bacteremic and non-bacteremic patients. Scand J Infect Dis.

[B3] Chalasani NP, Valdecanas MA, Gopal AK, McGowan JE, Jurado RL (1995). Clinical utility of blood cultures in adult patients with community-acquired pneumonia without defined underlying risks. Chest.

[B4] Campbell SG, Marrie TJ, Anstey R, Ackroyd-Stolarz S, Dickinson G (2003). Utility of blood cultures in the management of adults with community acquired pneumonia discharged from the emergency department. Emerg Med J.

[B5] Perl B, Gottehrer NP, Raveh D, Schlesinger Y, Rudensky B, Yinnon AM (1999). Cost-effectiveness of blood cultures for adult patients with cellulitis. Clin Infect Dis.

[B6] Stevenson A, Hider P, Than M (2005). The utility of blood cultures in the management of non-facial cellulitis appears to be low. N Z Med J.

[B7] Pitetti RD, Choi S (2002). Utility of blood cultures in febrile children with UTI. Am J Emerg Med.

[B8] Velasco M, Martinez JA, Moreno-Martinez A, Horcajada JP, Ruiz J, Barranco M (2003). Blood cultures for women with uncomplicated acute pyelonephritis: are they necessary?. Clin Infect Dis.

[B9] Corbo J, Friedman B, Bijur P, Gallagher EJ (2004). Limited usefulness of initial blood cultures in community acquired pneumonia. Emerg Med J.

[B10] Theerthakarai R, El Halees W, Ismail M, Solis RA, Khan MA (2001). Nonvalue of the initial microbiological studies in the management of nonsevere community-acquired pneumonia. Chest.

[B11] Sturmann KM, Bopp J, Molinari D, Akhtar S, Murphy J (1996). Blood cultures in adult patients released from an urban emergency department: a 15-month experience. Acad Emerg Med.

[B12] Wing DA, Park AS, Debuque L, Millar LK (2000). Limited clinical utility of blood and urine cultures in the treatment of acute pyelonephritis during pregnancy. Am J Obstet Gynecol.

[B13] Pasternak EL, Topinka MA (2000). Blood cultures in pyelonephritis: Do results change therapy?. Acad Emerg Med.

[B14] Eisenberg JM, Rose JD, Weinstein AJ (1976). Routine blood cultures from febrile outpatients. Use in detecting bacteremia. JAMA.

[B15] Church DL, Hall P (1999). Centralization of a regional clincal microbiology service: The Calgary experience. Can J Infect Dis.

[B16] Laupland KB, Gill MJ, Schenk L, Goodwin D, Davies HD (2002). Outpatient parenteral antibiotic therapy: evolution of the Calgary adult home parenteral therapy program. Clin Invest Med.

[B17] Gibb AP, Hill B, Chorel B (1998). Comparative study of BacT/Alert FAN bottles and standard BacT/Alert bottles. Diagn Microbiol Infect Dis.

[B18] Laupland KB, Church DL, Mucenski M, Sutherland LR, Davies HD (2003). Population-based study of the epidemiology of and the risk factors for invasive Staphylococcus aureus infections. J Infect Dis.

[B19] Thanassi M (1997). Utility of urine and blood cultures in pyelonephritis. Acad Emerg Med.

[B20] Glerant JC, Hellmuth D, Schmit JL, Ducroix JP, Jounieaux V (1999). Utility of blood cultures in community-acquired pneumonia requiring hospitalization: influence of antibiotic treatment before admission. Respir Med.

[B21] Ramos JM, Masia M, Elia M, Gutierrez F, Royo G, Bonilla F (2004). Epidemiological and clinical characteristics of occult bacteremia in an adult emergency department in Spain: influence of blood culture results on changes in initial diagnosis and empiric antibiotic treatment. Eur J Clin Microbiol Infect Dis.

[B22] Epstein D, Raveh D, Schlesinger Y, Rudensky B, Gottehrer NP, Yinnon AM (2001). Adult patients with occult bacteremia discharged from the emergency department: epidemiological and clinical characteristics. Clin Infect Dis.

[B23] Ramakrishnan K, Scheid DC (2005). Diagnosis and management of acute pyelonephritis in adults. Am Fam Physician.

[B24] Morris AJ, Wilson ML, Mirrett S, Reller LB (1993). Rationale for selective use of anaerobic blood cultures. J Clin Microbiol.

[B25] Elhanan G, Sarhat M, Raz R (1997). Empiric antibiotic treatment and the misuse of culture results and antibiotic sensitivities in patients with community-acquired bacteraemia due to urinary tract infection. J Infect.

[B26] Shapiro NI, Wolfe RE, Wright S, Spears J, Bates DW (2005). Who needs a blood culture? A prospectivlty derived and validated clinical prediction rule. Acad Emerg Med.

[B27] Bates DW, Cook EF, Goldman L, Lee TH (1990). Predicting bacteremia in hospitalized patients. A prospectively validated model. Ann Intern Med.

[B28] Chirouze C, Schuhmacher H, Rabaud C, Gil H, Khayat N, Estavoyer JM (2002). Low serum procalcitonin level accurately predicts the absence of bacteremia in adult patients with acute fever. Clin Infect Dis.

[B29] Caterino JM, Scheatzle MD, Forbes ML, D'Antonio JA (2004). Bacteremic elder emergency department patients: procalcitonin and white count. Acad Emerg Med.

[B30] Liaudat S, Dayer E, Praz G, Bille J, Troillet N (2001). Usefulness of procalcitonin serum level for the diagnosis of bacteremia. Eur J Clin Microbiol Infect Dis.

